# Acceptance of Medical Artificial Intelligence in Skin Cancer Screening: Choice-Based Conjoint Survey

**DOI:** 10.2196/46402

**Published:** 2024-01-12

**Authors:** Inga Jagemann, Ole Wensing, Manuel Stegemann, Gerrit Hirschfeld

**Affiliations:** 1 School of Business University of Applied Sciences and Arts Bielefeld Bielefeld Germany

**Keywords:** artificial intelligence, skin cancer screening, choice experiment, melanoma, conjoint analysis, technology acceptance, adoption, technology use, dermatology, skin cancer, oncology, screening, choice based, trust

## Abstract

**Background:**

There is great interest in using artificial intelligence (AI) to screen for skin cancer. This is fueled by a rising incidence of skin cancer and an increasing scarcity of trained dermatologists. AI systems capable of identifying melanoma could save lives, enable immediate access to screenings, and reduce unnecessary care and health care costs. While such AI-based systems are useful from a public health perspective, past research has shown that individual patients are very hesitant about being examined by an AI system.

**Objective:**

The aim of this study was two-fold: (1) to determine the relative importance of the provider (in-person physician, physician via teledermatology, AI, personalized AI), costs of screening (free, 10€, 25€, 40€; 1€=US $1.09), and waiting time (immediate, 1 day, 1 week, 4 weeks) as attributes contributing to patients’ choices of a particular mode of skin cancer screening; and (2) to investigate whether sociodemographic characteristics, especially age, were systematically related to participants’ individual choices.

**Methods:**

A choice-based conjoint analysis was used to examine the acceptance of medical AI for a skin cancer screening from the patient’s perspective. Participants responded to 12 choice sets, each containing three screening variants, where each variant was described through the attributes of provider, costs, and waiting time. Furthermore, the impacts of sociodemographic characteristics (age, gender, income, job status, and educational background) on the choices were assessed.

**Results:**

Among the 383 clicks on the survey link, a total of 126 (32.9%) respondents completed the online survey. The conjoint analysis showed that the three attributes had more or less equal importance in contributing to the participants’ choices, with provider being the most important attribute. Inspecting the individual part-worths of conjoint attributes showed that treatment by a physician was the most preferred modality, followed by electronic consultation with a physician and personalized AI; the lowest scores were found for the three AI levels. Concerning the relationship between sociodemographic characteristics and relative importance, only age showed a significant positive association to the importance of the attribute provider (*r*=0.21, *P*=.02), in which younger participants put less importance on the provider than older participants. All other correlations were not significant.

**Conclusions:**

This study adds to the growing body of research using choice-based experiments to investigate the acceptance of AI in health contexts. Future studies are needed to explore the reasons *why* AI is accepted or rejected and whether sociodemographic characteristics are associated with this decision.

## Introduction

Skin cancers are the most common groups of cancers diagnosed worldwide, with more than 1.5 million new cases estimated in 2020 [1]. Melanoma is the deadliest form of skin cancer. Based on demographic changes, it is estimated that more than 500,000 new cases of melanoma and almost 100,000 deaths from melanoma should be expected worldwide by 2040 [1]. As melanoma case numbers are expected to increase in the future, high-cost treatments will continue to put a strain on the already overburdened health care budgets. To combat the rising mortality rate of melanoma, early detection is critical. Currently, the German national treatment guidelines [2] recommend skin cancer screening as a standardized full-body skin examination performed by dermatologists who have completed specialized training in the early detection of skin cancer. In addition, dermatologists should use dermoscopy to diagnose suspected skin cancer. Given the rising number of cases as well as increasing scarcity of trained dermatologists [3-5], there has been substantial research into the feasibility of artificial intelligence (AI) to augment or replace traditional skin cancer screening regimens [6].

AI describes machines (or computers) that mimic the cognitive functions associated with human thought, such as learning and problem-solving. These systems observe their surroundings and adopt action to reach their targets directly [7]. Further, AI has the ability to learn from images and subsequently provide an image-based diagnosis. Dermatology, as an image-based field of medicine, retains a dominant position in the AI evolution with the ability to classify skin lesions [8].

Research into the technical quality of AI-based skin cancer screening technologies has shown that these systems achieve detection rates that are on par or better than those of highly trained clinicians [9-13]. This highlights the great potential of AI for future skin cancer screening in the general population. As part of apps, AI systems offer immediate access to dermatological screening for all patients with mobile digital devices, enabling health care and treatment to be provided regardless of time and place and close to everyday life [6]. Thus, AI systems capable of detecting melanoma and nonmelanoma skin cancer could avoid unnecessary care, reduce health care costs, offer solutions to the increasing scarcity of clinicians, and reduce the waiting times for an appointment and for a diagnosis [3-5]. However, there is a risk that some melanomas will be missed and treatment delayed if the apps incorrectly reassure the user that their lesion is of low risk [14].

Although the technical quality has improved, there is also a growing awareness that patients do not generally accept the use of AI-based systems in health care settings. There is still no consistent definition of technology acceptance in the literature. Terms such as “acceptability,” “acceptance,” and “adoption” are often employed in this context, sometimes interchangeably. Dillon and Morris [15] defined user acceptance “as the demonstrable willingness within a user group to employ IT [information technology] for the tasks it is designed to support.”

Khullar et al [16] conducted an online survey to examine patients’ perspectives about applications of AI in health care, showing that 31% of respondents reported being very uncomfortable and 40.5% were somewhat uncomfortable with receiving a diagnosis from an AI algorithm that was accurate 90% of the time but incapable of explaining its rationale. Longoni et al [17] demonstrated that consumers are very hesitant about being examined by an AI system and consumers’ willingness to pay decreases when an equivalent service is performed by an AI system. Additionally, they concluded that patients’ perceived neglect of uniqueness leads to more resistance to medical AI [17].

Past research has also identified several factors that might impact patients’ preferences to use AI-based health care services. The European Commission [18] interviewed citizens of the 28 member states of the European Union (N=27,900) and concluded that younger participants with a high educational level are more likely to use online health care services. This finding was also replicated in oncology patients, where younger patients indicated higher acceptance of and a greater intention to use digital tools and apps to manage their cancer [19]. The European Commission [18] also found that the opinion on AI strongly depends on exposure to related information and knowledge. This relationship is also supported by a series of experiments showing that resistance to the utilization of medical AI is driven by the subjective difficulty of understanding algorithms [13].

Concerning skin cancer, previous research has shown that patients were generally reluctant to use AI-based systems in the field of dermatology. Snoswell et al [6] examined the consumer preference and willingness to pay for mobile teledermoscopy services in Australia using a discrete-choice experiment (N=199). They found that patients prefer a trained medical professional to be involved in their skin cancer screening and that patients are less willing to pay money for teledermatology [6]. However, Snoswell et al [6] did not take into account sociodemographic factors that may have had an impact on the patients’ decisions. In a multicenter clinical study assessing the performance of automated diagnosis of melanoma with a self-completion questionnaire (N=65), Fink et al [20] found that most patients agreed that computer-assisted diagnoses are trustworthy and may generally improve the diagnostic performance of physicians. However, participants rejected the idea of AI-based systems completely replacing physicians and instead strongly favored hybrid solutions in which diagnoses by a physician are supported by automated systems [20].

To date, only three studies have directly addressed the question of which factors are associated with patients’ preferences regarding AI-based skin cancer screening [21-23]. Ghani et al [22] studied public interest in teledermatology, which was found to be positively associated with a younger age, higher educational attainment, and higher household income. Chang et al [21] examined sociodemographic differences in teledermatology acceptability with a cross-sectional survey (N=13,996), showing that respondents who were interested in teledermatology were more frequently 18-39 years of age, men, college graduates, and tablet or smartphone users. Similarly, young age, male gender, a previous history of melanoma, and higher educational level were significantly associated with a more positive attitude toward skin cancer–related apps [23]. However, it is unclear whether these results from questionnaires can be replicated in choice-based experiments that rely to a lesser degree on introspection and are thus one step closer to actual behavior [24].

As described above, provider and costs for a skin cancer screening have high relevance for the user [6,16,17,20]. For this choice-based conjoint analysis, we further added the attribute waiting time for a diagnosis, because studies have shown a strong negative correlation between patient satisfaction and waiting time [25,26]. AI provides the opportunity to get a skin cancer screening immediately, without any waiting time [3]. Due to the shortage of medical professionals, waiting time for a skin cancer diagnosis is also an important attribute for the user [5,7].

The aim of this study was two-fold based on the following two research questions: (1) How important are the attributes provider, costs for screening, and waiting time for diagnosis for participants’ preference for skin cancer screening? (2) Are sociodemographic characteristics, especially age, systematically related to the relative importance scores of participants to the various attributes?

## Methods

### Study Design

This cross-sectional study used a choice-based conjoint analysis to examine the acceptance of medical AI for a skin cancer screening from the user perspective. Conjoint analysis is a quantitative marketing research method that quantifies the value consumers place on the attributes of a product [27]. Respondents are asked to make a choice between 2 or more different choice sets, where each set is described in terms of several predefined attributes, each with different levels. Given a sufficient number of choices per respondent, it is then possible to statistically estimate the importance of each attribute and level for the choice in terms of part-worth utilities. This method offers a behavioral approach and is less susceptible to social desirability and other biases [28].

**Figure 1:**
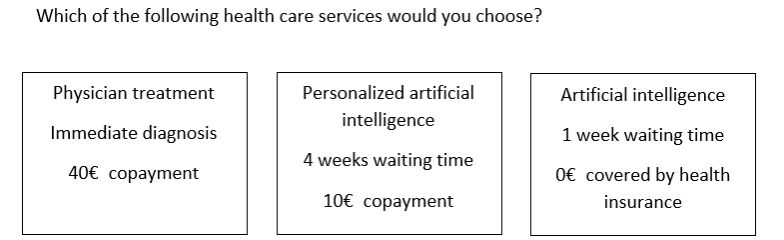
Example choice set (1€= US $1.09).

This study systematically manipulated three attributes (provider, cost, and waiting time) for a hypothetical skin cancer screening. Participants were presented with 12 different choice sets one after another, each consisting of three different modes of skin cancer screenings that were generated by combining different levels of the three attributes (see Figure 1 for an example). The choice sets were generated by the *conjointly* algorithm using default settings [29].

### Survey

Before the survey was conducted, it was tested with the “think-aloud” method by three volunteers to find out if there were any comprehensibility problems. For this purpose, the pretest participants had to speak their thoughts aloud while completing the survey [30].

The questionnaire started with informed consent, where participants were informed about the nature and scope of the survey and about the protection of their data. Before starting the questionnaire, participants completed the consent form and agreed to participate in the anonymous study. The participants then moved on to the choice-based conjoint task, which consisted of 12 different choice sets. The participant’s task for each choice set was to indicate the skin cancer screening that they most prefer (ie, they selected one of the three options as their preferred choice). After responding to the choice sets, participants were asked whether they had undergone a skin cancer screening in the last year and at which type of provider. Finally, the sociodemographic characteristics (age, gender, education, status, income) were assessed. Finally, the survey asked again whether the data could be used for analysis in anonymized form in case respondents changed their minds during the course of the survey and to filter out people who just wanted to “click through” without seriously answering the questions.

### Participants

Recruitment was based on a convenience sample through the social environment; individuals were asked to participate in the open voluntary survey shared with contacts via WhatsApp and Instagram. Standard procedures for conducting and reporting online surveys [31] were followed. Furthermore, conjointly’s default methods were used to identify and bar potential duplicate entries from the same user. Data were collected during the time period of September 29, 2022, through October 20, 2022.

The link to the survey was clicked 383 times by unique site visitors. Of these potential respondents, 126 (32.9%) people filled out the conjoint survey completely and gave their agreement for processing their data. In total, 220 (57.4%) respondents opened the link but did not complete the survey and another 33 (8.6%) people were disqualified from the study because they answered the survey several times. Three people (0.8%) did not give their agreement to process their data and a single respondent (0.3%) was excluded because the survey was answered too quickly. Respondents took an average of 4.7 minutes to complete the survey. Table 1 provides an overview of the respondents’ sociodemographic characteristics. There was a relatively equal proportion of participants identifying as male and female. The average age of the participants was 37.6 years and the median age was 29 years.

**Table 1:** Sociodemographic characteristics of participants sampled from September 29, 2022, to October 20, 2022 in Germany (N=126).

Variable			Participants, n (%)
**Gender**			
	Male	58 (46.0)	
	Female	67 (53.2)	
	Other	1 (0.8)	
**Education**			
	Still a student	1 (0.8)	
	School-leaving qualification	25 (19.8)	
	Vocational qualification	34 (27)	
	University degree	58 (46)	
	Doctorate	2 (1.6)	
	Other degree	3 (2.4)	
	Not specified	3 (2.4)	
**Employment status**			
	Elementary/high school student	2 (1.6)	
	University student	29 (23)	
	Apprentice	11 (8.7)	
	Employee	58 (46)	
	Civil servant	6 (4.8)	
	Self-employed	8 (6.3)	
	Not employed	1 (0.8)	
	Retired without income	9 (7.1)	
	Other	1 (0.8)	
	Not specified	1 (0.8)	
**Monthly income (Euro; 1€=US $1.09)**			
	<250	5 (4.0)	
	250-499	8 (6.3)	
	500-999	17 (13.5)	
	1000-1499	12 (9.5)	
	1500-1999	13 (10.3)	
	2000-2999	26 (20.6)	
	3000-3999	16 (12.7)	
	4000-4999	6 (4.8)	
	>5000	13 (10.3)	
	Not specified	10 (7.9)	

### Ethical Considerations

Our online study was conducted in accordance with the American Psychological Association’s Ethical Principles of Psychologists and Code of Conduct. In particular, data collection was anonymous; harmless to participants; and did not involve deception, injure, or place participants under high levels of physical or emotional stress. In line with 2023 guidelines of the German Research Foundation, formal ethical approval was not required because our study did not include aspects that would necessitate a statement, per subsection two of the "Information on proposals in the field of psychology" [32]. Informed consent was obtained from all participants after the purpose of the study and the data collection were outlined in the survey introduction. Participants indicated their consent by clicking a button. Study data and identifiers were anonymized during the data collection and data analysis to maintain confidentiality. No compensation was awarded to participants.

## Results

Table 2 provides an overview of the relative importance values of the attributes and part-worth values of each level for each attribute as determined by conjointly to answer the first research question [29]. A treatment by a physician that is completely compensated by insurance and has no waiting time emerged as the most preferred mode of treatment. Overall, *provider* was the most important attribute, followed by *costs* and *waiting time*. For all attributes, we found two levels with part worths around zero and one positive and negative level. For *provider,* the physician had a positive part worth and the AI system had a negative part worth, while both the personalized AI and teledermatology had near-zero part worths. For *waiting time*, immediate results had a positive part worth and a 4-week wait had a negative part worth, while a 1-day and 1-week wait had similar near-zero part worths.

**Table 2:** Part-worth and relative importance values of the attributes.

Attribute			Part worth (95% CI)		Relative importance, % (95% CI)
**Provider**					38.6 (35.3 to 41.6)
	AI^a^	–0.15 (–0.17 to –0.13)			
	Personalized AI	–0.06 (–0.08 to –0.04)			
	Physician treatment	0.21 (0.19 to 0.24)			
	Electronic consultation with physician (teledermatology)	0.005 (–0.01 to 0.02)			
**Costs for screening^b^**					31.6 (29.0 to 34.0)
	0€ (completely covered by health insurance)	0.15 (0.13 to 0.16)			
	10€ copayment	0.06 (0.06 to 0.07)			
	25€ copayment	–0.03 (–0.04 to –0.03)			
	40€ copayment	–0.18 (–0.19 to –0.16)			
**Waiting time for diagnosis**					29.8 (27.2 to 32.3)
	Immediate	0.10 (0.09 to 0.11)			
	1 day	0.082 (0.07 to 0.09)			
	1 week	–0.004 (–0.01 to 0.003)			
	4 weeks	–0.18 (–0.20 to –0.17)			

^a^AI: artificial intelligence.

^b^1€=US $1.09.

**Figure 2:**
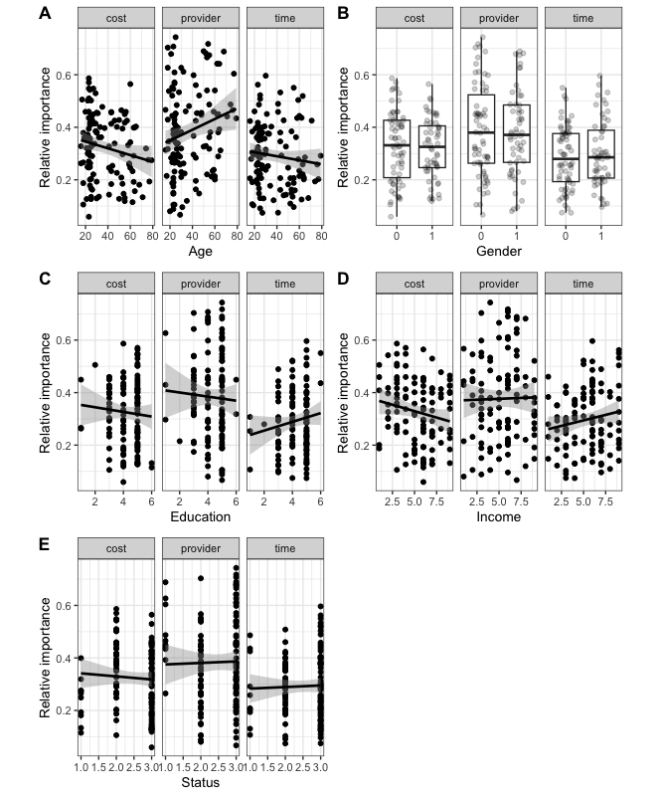
Relationship between relative importances and sociodemographic characteristics: (A) age, (B) gender, (C) education, (D) income, (E) employment status.

Figure 2 shows an overview of the relationships between sociodemographic characteristics and the relative importances to answer the second research question. We found a medium-sized positive relationship between age and provider. In addition, there were two nonsignificant trends. The first indicated an inverse relationship between age and the importance of costs and the second indicated an inverse relationship between income and the importance for costs. All other importances were not systematically related to sociodemographic variables (Table 3).

**Table 3:** Correlation coefficients (Spearman ρ) for the importance values.

Sociodemographic characteristics			Relative importance				
			Provider		Costs for screening		Waiting time for diagnosis
**Age**							
	Coefficient	0.21^a^		0.17		0.11^a^	
	*P* value	.02		.05		.25	
**Gender**							
	Coefficient	–0.003		–0.03		0.04	
	*P* value	.97		.71		.60	
**Education**							
	Coefficient	–0.04		–0.06		0.07	
	*P* value	.64		.45		.45	
**Employment status**							
	Coefficient	0.05		–0.11		–0.004	
	*P* value	.53		.22		.96	
**Income**							
	Coefficient	0.02		–0.17		0.11	
	*P* value	.81		.07		.24	

^a^Pearson correlation coefficient.

## Discussion

The aim of this study was to determine how important the attributes provider, costs, and waiting time are for users’ preference for skin cancer screening and to investigate whether sociodemographic characteristics, especially age, are systematically related to participants’ individual importances. We found that provider was as equally important a factor for participants’ decisions as cost and waiting time. While a physician was the most preferred level of this attribute, AI-based treatment was disliked and a personalized AI had the same value for participants as teledermatology. Concerning the relationship between sociodemographic characteristics and relative importances, we found that only age showed a reliable positive association to provider, in which younger participants place less importance on the provider than older participants. In the following, we discuss these findings in turn before discussing the limitations of the study and providing a general outlook.

Regarding the role of the provider in users’ decisions, other studies underline our results that patients exhibit hesitant behavior toward medical AI. Patients would rather not have a treatment than be examined by an AI system, even if the AI system shows the same or better accuracy as a physician [17]. However, the same study also found that patients prefer personalized AI over nonpersonalized AI. Similarly, earlier discrete-choice experiments [6] as well as surveys [33] found that patients prefer a trained medical professional to be involved in their skin cancer screening; 41% of respondents were open to using AI as a standalone system for skin cancer screening and 94% were open to using it as a support system for physicians [33]. Together, existing studies indicate that personalized AI and teledermatology are generally more accepted than nonpersonalized AI for skin cancer screening, while the physician remains the most preferred option.

Concerning the impact of age differences on the acceptance of AI in dermatology, our findings also support some earlier results [21-23]. Higher interest in using teledermatology [21,22] and in using skin cancer–related apps [23] was associated with younger age. The results of cross-sectional studies back up our findings from the choice-based conjoint analysis. Based on these trends, it is possible to imagine that the acceptance of AI in skin cancer screening will rise in the future due to the aging of digital natives and their increased acceptance of AI.

Regarding income and educational factors, our findings do not align with those of previous studies. Ghani et al [22] concluded that higher education attainment and a higher household income increased the interest in using teledermatology. Chang et al [21] came to similar conclusions, indicating that college graduates showed the greatest interest in teledermatology. In addition, Steeb et al [23] showed that a high educational level was associated with a positive attitude toward skin cancer–related apps. While we were not able to show significant relationships to income and educational background, the smaller sample size in this study compared to those of earlier studies might explain this inconsistency.

Previous studies also identified gender differences in the acceptance of AI in skin cancer screening. Chang et al [21] came to the conclusion that men are more likely to use teledermatology than women. Steeb at al [23] found similar results in which male gender was significantly associated with a positive attitude toward skin cancer–related apps. However, the gender difference that was reported in earlier studies was not visible in our data. Again, this might be a factor of sample size, but it also might also be that these gender differences reported in earlier questionnaire studies reflect differences in the technology self-concept [34] rather than actual preferences.

Several aspects must be considered in interpretation of our findings. First, the sample was not randomly selected but was based on a convenience sample. While a wide range of recruitment means were used, the results are likely not generalizable to the general public but rather more specific to highly educated young adults. Further research is needed with the target group. Although the sample size may not seem particularly large, sensitivity analysis showed that this sample size was in fact sufficient to detect a medium-sized correlation (*r*=0.28) with a power of 90% and error rate of 5%. Second, some participants contacted us about the meaning of the attribute *waiting time* because they were unsure whether this pertained to the waiting time for a diagnosis or the waiting time for an appointment. Future studies should make this distinction more explicit to study possible differential effects of these two types of waiting times.

Taken together, we believe that this study adds to the growing body of research using choice-based experiments to investigate the acceptance of AI in health contexts. This approach offers additional insights and is less susceptible to social desirability and other biases [35]. However, the choice-based conjoint analysis only allows studying a small number of potential attributes at a time [24]. Because we included personalized AI as a level for the attribute provider, our study adopted the findings of Longoni et al [17] that personalized AI increases patient acceptance. In addition, we examined factors that may have an impact on patients’ decision-making following the study of Snoswell et al [6].

For the future, it could be interesting to add “AI as a physician support system” to the choice set [33]. It might also be interesting to find out whether patients who perceived themselves as more individualized are less accepting of AI [17]. Additionally, it could be interesting to explore whether specialized knowledge about AI systems would increase patient acceptance [13] and which other factors might have an influence on patients’ acceptance. Ideally, this would not only rely on correlational evidence as used here but also on experimental evidence that shows how preferences and importances may be altered. The variables such as income and educational background cannot be manipulated easily. Nevertheless, we believe that the magnitude of these effects provides some benchmarks for future studies that aim to use experimental methods to alter preferences.

In summary, while there have been technological advances in the effectiveness of AI for supporting skin cancer screening and health care more generally, we believe that the true potential of AI systems can only be realized if patients’ needs and demands are taken into account.

## Abbreviations

AI: artificial intelligence

## Data Availability

The data sets generated and analyzed during this study are available in the zenodo repository [36].
